# Five Genes: Key Qualities for Selecting Residents with High Potential

**DOI:** 10.15694/mep.2018.0000031.1

**Published:** 2018-02-05

**Authors:** Vijay Rajput

**Affiliations:** 1 Ross University School of Medicine

**Keywords:** Resident Selection, Student quality

## Abstract

This article was migrated. The article was marked as recommended.

During my time as program director, I have reviewed hundreds of residency applications, familiarized myself with the application process, and discovered five characteristics or “genes” that contribute to the success of a future resident. With the advent of an online residency application system, filters tend to focus on more quantitative data like test scores, which may not capture an applicant’s true ability to be a successful resident. Applicants’ letters of recommendation from both deans and physicians are also increasingly becoming harder to interpret due to diction that is either too hard to understand and/or has the potential to come off negatively. Personal statements, while attempting to capture an applicant’s valuable experiences and beliefs, tend to fall short because most applicants either share too strong of political or religious views or do not share enough substance to make a statement truly “personal.” Beyond these typical application materials, how a student communicates with others outside of an interview is key for determining a student’s true professional behavior. This communication could be in the form of scheduling, rescheduling, or cancelling interviews or how the student interacts with the residents at the informal dinner the night before the interview. Along with professional communication, five “genes” that make for a successful resident, as mentioned above, are passion for the profession, intellectual curiosity and investment, work ethic, ability to be a team player, and empathy. If armed with these qualities, an applicant can turn into a successful resident in no time.

## Introduction

In my role as a medical educator over the last 20 years, I have interviewed hundreds of medical students applying for residency in Medicine. The most time- consuming part of the interview process begins with screening student files. These files include quantitative and qualitative data about each student, specifically their application, Curriculum Vitae, transcripts, letters of recommendations, Medical Student Performance Evaluation (MSPE), and personal statement. I clearly remember taking home these files on a Friday and screening them while watching the Sunday morning news. Over the past decade, with the advent of the Electronic Residency Application System (ERAS), residents’ files and the review process has changed since the applications are now better organized and easier to screen. The current ERAS software is a filtering system that residency programs can use to screen applicants without having to perform a full review of each application. Filters can be based on various application features such as examination failures, origin of medical school, year of medical school graduation, and many other epidemiological characteristics. These filters create new types of discrimination and barriers for students, since it is not necessary to review all the unique characteristics from each application. As a result of these filters, many student files are never reviewed by faculty because they have been pre-emptively removed. This process creates a high degree of discrimination and damage for those applicants who have had some setbacks in their files, i.e., a failure in standardized tests like the United States Medical Licensing Examination (USMLE). This detrimental filter system has the power to “filter out” excellent applicants who will never get an opportunity to practice the specialty they are passionate about because of one or two setbacks. A poor score indicates poor knowledge, but poor knowledge can be improved with practice and guidance over time. However, an outstanding score does not automatically indicate the student will make an excellent resident and have sound practical judgment. We, in the medical field, are fixated on the past performance of medical students and we don’t look deeper into their ability to recover from mistakes and uncover their true potential. In the business world, applicants with high potential possess the strong capacity to grow and succeed throughout their careers within their respective organizations. ^(1)^ I believe that these five genes can help students reach their highest potential.When taking a closer look at the files themselves, one part that is difficult to interpret is the letter of recommendation.

Most programs require that students submit three recommendation letters from physicians they have recently worked with, such as the clerkship director in the specialty to which they are applying to. The problem arises partly because faculty members do not have an ethical obligation to write a constructive assessment of students in their recommendation letters. It can be difficult to make sense of many of these letters as they often contain descriptive words and language that is complex and hard to understand. For example, the differences between “outstanding,” “excellent,” “very good,” and “good” can be wide, since each faculty member and reviewer can interpret these words differently. The words “solid” or “improving” are words that can potentially affect an application negatively, but the letter writer may not be thinking these words are deal breakers.

In the past, when I served as program director, I found it very challenging to decode the implicit meanings for key adjectives in letters of recommendation. On the other hand, many program directors rely more on the quantitative qualities of the application, like the objective numbers from standardized examinations. Numbers do reflect a candidate’s cognitive ability to synthesize and analyze medical knowledge. However, these tests do not identify some of the core attitudes and behaviors needed to make good judgment and treatment decisions for patients. As letters of recommendation slowly move from narrative to standardized, it becomes difficult to interpret what makes an ideal candidate stand apart from others. ^(2)^ Students’ numbers also highlight this feeling that when I am reviewing 50-100 files at a time, many student files begin to look alike. There are often only minor differences in scores and students’ performances in courses and clerkships. The letter from the dean’s office and other recommendation letters are often bland. The similar language contained from letter to letter is generally not helpful in picking out a “gem” or “diamond in the rough” from all the other potential applicants. I remember, many times, inviting a student to interview based purely on his or her medical school, geographical location of birth, or a life journey in a specific part of the United States. Additionally, on many occasions, I invited students based on who wrote their letter of recommendation. For example, I once invited a student for an interview because one of their recommendation letters was written by a great speaker I had heard at a national medical education meeting. This phenomenon is common, because for the past few years, I have met with colleagues and developed professional and academic relationships with them at both the regional and national level. As a result, I am continually searching students’ files for personal connections to my professional life. Throughout my career, I must have read thousands of personal statements written by students applying for medical school and residency and I have never found these particularly useful in determining their potential as future physicians. However, the personal statement is a helpful conversation piece during the actual interview itself. I usually read each applicant’s personal statement just prior to the interview to identify or match their qualities and personal views towards the medical profession and health care system. The issue I have with personal statements is that I have never truly “fallen in love” with a personal statement. Sometimes, I become nervous hiring a student who shares strong political or religious views through their statement. Rarely, are these personal statements truly “personal.” Often, students don’t seem to feel comfortable openly sharing about their personal lives to those who are going to hire them for the residency. With students experiencing pressure to have a personal statement “perfect,” some turn to plagiarism. This evidence of plagiarism is found across all specialty programs and medical school types, even with students with significant honors in their files. ^(3)^

A student’s life stays busy between the time they receive invitations for interviews and when they come to the interviews themselves. During this time, the promptness and tone of written and verbal communication between administrative staff and students can, at times, give more insight into a student’s work ethic and communication skills than during the interview itself. During the last years of their education, students mastered how to behave appropriately in a structured and observed learning and assessment environment. Yet, I always wondered how students would behave when no one is watching them or when they are communicating with people who have no direct reporting responsibility or authority over them. The initial scheduling, possible cancellation and rescheduling of an interview during the process can shed some light on student’s ethical and professional behaviors. Many times, this cumulative communication leading up to the interview can be very telling, and I would rely on my program coordinator to alert me if there are any glaring, red flags in the communication. Especially during the last few months of the interview season, many students cancel their interview the night before, which most program directors are aware of. Students who cancel their interviews, especially at the last-minute lack proper judgment and effective decision-making skills. I wish there was some magical way to identify the students, ahead of time, who will cancel their interviews last minute, but that magic is unlikely. Surprisingly, interview communication between the programs and applicants has been challenged and discouraged among few specialties. Additionally, there has been match violation reported among a few minority of programs. ^(4)^ Interview seasons remind me of a time when I was reading an article in a business journal that said if one wants to know someone’s true personality, he or she should invite them over for dinner. Our residency program arranges a dinner with student applicants and current residents at a local restaurant the evening before the actual interview. During this time, our current residents have informal conversations with the students to pick up on informative body language cues and vibes of the students’ interpersonal communication and professional attitudes. I learned my lesson the hard time, when I ignored my resident’s concern about a student’s behavior and interpersonal communication during a dinner. It was rare that I could pick up on the fine nuances of individual students’ behavioral and communication issues just from their letter of recommendations or MSPE. Plus, interview days are all well-structured to showcase specialties and individual programs, so there is not much attention on nuances in behavior. Most interview days include an overview done by the program directors and chief residents while one or two faculty members interview each student. I always wonder if applicants retain any of the information they learn throughout the day since these primary care programs invite 10-30 students at a time and run all day long. My interviews usually focus more on the student’s lives and their relationships with their co-workers rather than on their academic performance. I always enjoy trying to identify a few characteristics or attitudes based on how they handled specific situations in their lives. On many occasions, an applicant will ask what I am looking for in an ideal or “perfect” resident. I jokingly respond that there are “Five Genes” I wish I could obtain from the DNA of every resident’s cheek swab that I believe make up traits for a successful resident. Here are the five genes that can make students into competent residents and practicing physicians.

**Gene 1: Passion for the profession:** It took me a long time to understand the true meaning of passion. I think most of us get confused between liking, having an interest in something and true passion. It is never that easy to identify who has a true passion for the profession. At times, I can pick up cues by asking a few questions about their most frequent activities. Many times, the amount of research and scholarships that a student has recorded in their CV is directly proportional to the demand for their specialty. Several studies over the last decade described falsified information or padding of resumes about research papers documented in students’ ERAS applications. ^(5)^ Many times, I have tried to connect the dots backwards in the files to identify the passion and have been wrong on several occasions. I have seen students who never had the opportunity to fulfill their passions suddenly flourish in the right environment. This makes me wonder if any student can develop passion once they are exposed to nurturing mentors and a safe environment. I have tried to identify a sparkle in students’ eyes while they respond to my questions. I have discussed current challenging issues in their area of interest to see if they are knowledgeable about it. It is hard to differentiate between the enthusiasm during an interview and true passion for the profession. There are many times I wish I could judge passion right away from both the file and interview. I know the best learning occurs when there is a passion for the profession and this true passion is a must for unstructured and asynchronous learning during residency.

**Gene 2: Intellectual Curiosity and Investment:** I am not sure I truly know how to recognize curiosity in each individual. This ability would be useful because it would give me a sense on which types of students will enjoy thinking and will go the extra mile to understand things when information or knowledge does not make sense to them. In psychological science, intellectual curiosity has been described as a “need for cognition” or the tendency for an individual to engage in and enjoy thinking. When a patient’s history and physical examinations do not match the laboratory and radiological tests, innate curiosity will help raise new questions necessary to reach a diagnosis. This clinical, bedside curiosity enables students and residents to think outside of the box to come up with solutions to patients’ ailments. In an era of point of care information technology, it has become far easier to access information in real time. The “why”, “how”, and “what” questions related to patient care are not raised very often anymore as a result. In medicine, it is very easy to follow the patterns and routine protocol as expected from residents. It has become much harder for this process to occur in the era of chronic use of “Up-to-date.” My biggest criticism for the use of Up-to-date is that I do not see it as true intellectual curiosity. Rather, the process is similar to ordering fast food and receiving instant gratification, instead of slowly and conscientiously following a recipe with different ingredients. When a student searches PubMed or Google Scholar and discusses questions with senior faculty, he or she will always come across a paper or information of which they were not previously aware of. This does not often happen with Up-to-date or textbooks. Such connections create new knowledge and innovation. We do not reward such intellectual curiosity at the bedside and this attitude is difficult to assess during the residency interview process.

**Gene 3: Work ethics (The extra mile concept):** I have always joked with my students and residents that the person who really knows their work ethic best is their mother. If I had permission to speak to mothers, they would give me unbiased answers. Work ethic is comprised of many attitudes and behaviors, including reliability, accountability, initiative, and a positive “can do” attitude. A student’s application or resume does not offer much insight into work ethic. During the clerkship years, a student can score perfectly on their examinations and show a passion and desire to excel in the field without having any real work ethic. Occasionally, a student can even hide a deficiency in work ethic by doing extra work or simply impressing the right supervising faculty. Commercial tests have been developed and used in business for assessing an employee’s work ethic; however, residency programs do not routinely use these tests. Increasing responsibility outside of course work can give some insight into a student’s work ethic. I still have a hard time identifying students who will go the “extra mile” for patients, when necessary. I also believe this gene is hard to discover when reviewing files and performing interviews.

**Gene 4: Team player:** I find qualities of a team player to be the most critical for residency. Working side by side with nurses, social workers, case managers and other health care professionals can be challenging, if the student has not developed their team skills during their undergraduate and medical school years. From the first day of internship to the entirety of a medical career, it is all about team work. My best team player residents actually enjoy the busy internship hours since students rarely have the opportunity to demonstrate their ability to work in a team during their course work and clerkships. Standard assessment tools used in medical school do not routinely assess for team building or team player skills, as these tools are focused on individual skills, attitudes, and behaviors. I have seen students with high scores on both USMLE examinations and clerkships who turn out to be poor team players. There are occasions when I can extract team building skills from prior work outside of medicine plus a few specific questions about their understanding of a successful team and being an effective team player. Some interview questions that may help include the following: tell me when you worked with a difficult group, what made the group difficult, how did you handle that situation, when was the last time you had to work out a disagreement with your peers or colleagues, give me some examples when people sought your advice and when you sought others out for advice, and are you happier working with data or working with other individuals. All these questions may or may not help to identify the qualities of a team player.

**Gene 5: Empathy:** A recent article by Daniel Goleman in the Harvard Business Review described the Empathy Triad. ^(6)^ He describes cognitive empathy as well as emotional empathy and empathetic concern to be critical qualities for leadership. Emotional empathy and empathetic concerns are now being taught in every medical school as a part of communication skills training and are assessed in the USMLE clinical examination. Many students can learn to create artificial emotional empathy and empathetic concern during this structured assessment environment. I have seen many students who demonstrate poor bedside emotional empathy, yet lack even a single comment about that in their files. I rely on my staff and residents to provide verbal or written alerts regarding such lapses when applicable. Over the years, I have hired arrogant and competent jerk type residents purely based on their outstanding scores and the halo effect they have created with their illusory knowledge superiority among their peers and supervising faculty. Cognitive empathy is the ability to relate to others or understand their viewpoints and reasoning. This quality is critical for patient care in our current complex collaborative practice. A student may not need to show much cognitive or intellectual empathy since many times students are competing with peers to “show off” their knowledge. Demonstrating cognitive empathy creates a safe and conducive learning and teaching environment. Additionally, an ability to understand the limitation of others’ knowledge is important for intellectual debates during rounds, conferences and morning reports. Cognitive empathy encompasses evaluation of both sides of an argument. Unless we deliberately force ourselves to consider alternatives by listening to others’ points of view, we are more prone to recalling evidence consistent with our fixed proposition. It is hard to overcome this mental weakness known as confirmation bias. We can try to be aware of this limitation and compensate for in-born tendencies towards self-serving and biased recollections through discipline and the cultivation of cognitive empathy.

**Conclusion:** Along with these five genes, there are many other attitudes and behaviors that are important for physicians. The debate between hard science and soft science is not going to die down easily. Intelligence and conscientiousness are mutually dependent, although many times, one can create a halo effect on one or the other. It has been debated that the “less” competent individual may become more conscientious to compensate for their lower level of cognitive ability or vice versa as the competent cardiothoracic or neurosurgeon can “afford” to be less conscientious. I hope that medical educators in the future focus more on assessing the “Five Genes” and therefore provide more meaningful assessments of these attitudes in their letters of recommendation and dean letters and contribute to more holistic residency applications.

## Take Home Messages

• A student who is applying for residency should be aware of how they communicate with program directors, faculty, residents, nurses, and anyone else outside of their interview day that could potentially report back to the interviewer. It is important for students to conduct themselves professionally at all times.
• Failures or poor scores in standardized testing does not fully identify students’ abilities to be successful residents nor does it capture their abilities to make effective judgment and treatment decisions for patients.
• It is important for faculty who write letters of recommendation for students to be very clear with their choice of language. Do not use words like “solid” or “improving” because it can give the admissions committee a negative view on the applicant. Give specific examples as to why this applicant would make a successful resident and go beyond just using words like “good” or “excellent.”
• It is important for applicants to feel truly passionate for the profession they are choosing. If an applicant is passionate, it will be clearly demonstrated throughout the interview day. Applicants should also be intrinsically motivated to learn new things every-day and be proactive in discovering the answers to concepts that they find tricky.
• Applicants should also go the extra mile and demonstrate a solid, consistent work ethic. They should demonstrate skills that highlight them as a team player and learn how to be empathetic, especially during clinical bedside training.

## Notes On Contributors

Dr. Vijay Rajput, MD is a Professor and Chairman of Medicine at Ross University of School of Medicine (RUSM). He also serves as Associate Dean for Clinical Sciences and Director for Office of Student Professional Development for RUSM at Miramar, Florida, USA.
