# Supplementary material for: Five Genes: Key Qualities for Selecting Residents with High Potential
Source: MedEdPublish (2016). 2018 Feb 5;7:31. [Version 1] doi: 10.15694/mep.2018.0000031.1 (PMC10711997; doi:10.15694/mep.2018.0000031.1)
Supplement: Peer review history

# Reviewer report: Reviewer response for version 1

**DOI:** 10.21956/mep.19507.r28845

**Published:** 2018-03-10

**Authors:** Mohamed Al-Eraky

**Affiliations:** 1 University of Dammam

This review has been migrated. The reviewer awarded 5 stars out of 5 A very nice reflective paper that represent years of experience in selection processes in residency programs. I agree with the points of the author, and further advocate that they may be more than five, as Trevor mentioned. The only reservation is that we don't want to be too judgemental. Meaning, acknowledging these 5 qualities as genes make them too hard to change. While some can be taught and assessed over a period of time. It's like raising a kid, but perhaps more challenging. Also, what about ranking or setting priorities. Do all of these 5 genes have the same value for all disciplines of residency. Perhaps, we need to validate this on the ground from the perspective of program directors of various specialities. Then we need to incorporate those core values into potential applicants. Instead of letters from someone or by themselves, we may ask them to provide evidence for these core values. A good food-for-thought article. Thank you.

Reviewer Expertise:

NA

No decision status is available

# Reviewer report: Reviewer response for version 1

**DOI:** 10.21956/mep.19507.r28844

**Published:** 2018-02-14

**Authors:** Subha Ramani

**Affiliations:** 1 Harvard Medical School, Brigham and Women's Hospital

This review has been migrated. The reviewer awarded 5 stars out of 5 Being a core residency program faculty and having interviewed several candidates over theist decade, I identify with the principles in this paper. Much work is being done on student education, not much on residency / postgraduate education. In medicine residency, we often see superb candidates already enmeshed in their future subspecialty interests and interview conversations veer off into their research or scholarship.This paper is well written, much needed for all educators who work in postgraduate training and participate in selection. While the author has acknowledged that some of these qualities, so essential, are difficult to 'measure', I completely agree with the importance of these genes. Perhaps we should be conducting interviews at the bedside to see how they interact with patients, families, nurses etc?Shiphra Ginsburg's and colleagues have extensively researched faculty comments on evaluations. comments are often vague and a reader has to decode what it actually means about the learner performance. Similarly, applicant letters are encoded from the sources and need to be decoded by interviewers.I hope the author or others continue this work by research in this area of residency selection.

Reviewer Expertise:

NA

No decision status is available

# Reviewer report: Reviewer response for version 1

**DOI:** 10.21956/mep.19507.r28843

**Published:** 2018-02-08

**Authors:** P Ravi Shankar

**Affiliations:** 1 American International Medical University

This review has been migrated. The reviewer awarded 4 stars out of 5 I enjoyed reading this well written and interesting manuscript. I agree with the author that the use of filters in the electronic application system for residency may be unfair in a number of ways. Graduates from certain medical schools, from certain geographic regions and those who had unfavorable scores in some standardized exams are automatically excluded. Students over the course of their undergraduate medical education learn to perform well under standardized assessment situations and this performance may not be indicative of how they will perform under real life circumstances. This has been mentioned in other papers published in MedEdPublish. The increasing emphasis on numbers and unbiased measures has led to an overemphasis on standardized exam and other scores. Also course directors and Deans tend to produce a nearly standardized letter for students due to various circumstances including a shortage of time. Also the increasing pressure to produce a powerful personal statement may be a reason why some students resort to plagiarism. Many residency directors are more favorable toward students who have worked in their hospital or toward students recommended by persons they know. This may disfavor some applicants. The author has summarized his recommendations very well in the take home messages section. Educators involved in the residency selection process will find this manuscript of special interest. But this interesting manuscript should also appeal to all those with an interest in medical education.

Reviewer Expertise:

NA

No decision status is available

# Reviewer report: Reviewer response for version 1

**DOI:** 10.21956/mep.19507.r28846

**Published:** 2018-02-08

**Authors:** Trevor Gibbs

**Affiliations:** 1 AMEE

This review has been migrated. The reviewer awarded 4 stars out of 5 An interesting paper to read; a personal reflection on how we effectively select postgraduate students into our training programmes. I am not sure that there are just 5 essential "genes" but would agree with the author that the five described are important if not very difficult to assess. As common with reflective papers like this, the answers don't jump out of the page, but this paper does make you think and therefore I feel it is a useful paper to read for all those involved in any form of student selection.

Reviewer Expertise:

NA

No decision status is available
